# Development of molecularly imprinted polymers for the removal of levofloxacin

**DOI:** 10.1186/s13065-025-01677-x

**Published:** 2025-11-24

**Authors:** Rashid Mahmood, Showkat Ahmad Bhawani, Syed Rizwan Shafqat, Rachel Marcella Roland

**Affiliations:** 1 Faculty of Resource Science and Technology, Universiti Malaysia Sarawak, Kota Samarahan, Sarawak, 94300 Malaysia; 2 Department of Chemistry, University of Sialkot, Sialkot, Pakistan

**Keywords:** Levofloxacin, Molecular imprinted polymer, Batching binding, Adsorption, Rebinding

## Abstract

The extensive use of antibiotics and their persistence in the environment make them emerging pollutants of global concern, posing serious risks to ecosystems and public health. Among them, the broad-spectrum fluoroquinolone antibiotic levofloxacin (LEV) is widely prescribed for bacterial infections and has frequently been detected in freshwater systems and environmental matrices. Its presence is linked to the development of antibiotic resistance and ecological toxicity, underscoring the urgent need for efficient removal strategies. In this study, levofloxacin-imprinted polymers (LEV-MIPs) were developed using a precipitation polymerisation method. A set of nine LEV-MIPs was synthesised using three solvent combinations: ethanol: acetonitrile, ethanol: dimethyl sulfoxide, and ethanol: carbon tetrachloride. Methacrylic acid (MAA) served as the functional monomer (1–3 mmol), ethylene glycol dimethacrylate (EGDMA) as the cross-linker (16 mmol), and azobisisobutyronitrile (AIBN) as the initiator (0.1 mmol). Among them, two formulations (LEV3-MIP and LEV6-MIP) demonstrated superior removal efficiency. Structural and thermal characterisation by FTIR, SEM/EDX, and TGA confirmed successful polymer synthesis, with surface analysis revealing spherical, monodispersed particles of ~ 1.5 μm. Batch adsorption assays showed removal efficiencies of 97.85% (LEV3-MIP) and 99.15% (LEV6-MIP) under optimised conditions (15 ppm initial concentration, 0.3 mg dosage, pH 7, and 90 min and 60 min contact time, respectively). Both polymers exhibited high imprinting factors (3.081 and 3.359) and excellent reusability, with minimal efficiency loss of only 2.7% and 2.09% after ten adsorption–desorption cycles. These results highlight the strong potential of LEV-MIPs as cost-effective, selective, and reusable materials for mitigating antibiotic pollution.

## Introduction

Molecularly imprinted polymers (MIPs) are synthetic materials designed to mimic natural recognition systems through the formation of specific binding cavities complementary to the size, shape, and functionalities of target molecules. Their high stability, cost-effectiveness, and simple synthesis strategies have made them attractive alternatives to natural receptors in a variety of applications, including biosensing, separation, diagnostics, environmental monitoring, and drug delivery [1–3]. Recent advances in imprinting technologies have further enhanced the affinity and selectivity of MIPs, enabling their use as antibody mimics and separation tools in increasingly complex biological and environmental matrices [4–7]. In environmental remediation, MIPs represent an advanced class of adsorbents that overcome the limitations of conventional sorbents such as poor selectivity, competitive adsorption, and limited reusability [8–10]. Antibiotics, particularly fluoroquinolones, have emerged as critical pollutants due to their persistence, accumulation, and contribution to antibiotic resistance genes (ARGs) in the environment [11, 12]. Among them, levofloxacin (LEV), a widely used broad-spectrum fluoroquinolone, is frequently detected in water bodies due to incomplete metabolism, industrial discharge, and improper disposal [13, 14]. Its high stability and poor degradability exacerbate risks of environmental accumulation, oxidative stress in aquatic organisms, and the spread of resistant bacteria [15–17]. Conventional techniques such as coagulation, flotation, and adsorption using non-specific sorbents have been employed for antibiotic removal [12, 18]. However, these methods often lack specificity, suffer from high operational costs, and may generate secondary pollutants. Adsorption remains a practical approach due to simplicity and low cost, but its effectiveness is restricted by competitive adsorption and poor reusability.

To address these challenges, the design of MIPs specifically tailored for antibiotics offers superior selectivity and regeneration potential [19, 20]. A further dimension that has gained importance is the greenification [21] of MIP synthesis. The use of environmentally benign solvents, renewable monomers, and energy-efficient polymerisation strategies is increasingly being explored to align MIP development with green chemistry principles [22, 23]. Incorporating sustainability into MIP design is especially critical when addressing large-scale water treatment applications. Due to their repeatability, high selectivity, stability and reproducibility, molecularly imprinted SPR sensors help in an early diagnosis of a disease which is caused by the application of pesticides [24]. The present work focuses on the development of levofloxacin-imprinted polymers (LEV-MIPs) synthesised via precipitation polymerisation using methacrylic acid (MAA) as the functional monomer and varying solvent systems (ethanol: acetonitrile, ethanol: DMSO, and ethanol: CCl₄). By optimising the monomer-to-template ratio and solvent composition, we aim to achieve MIPs with enhanced selectivity, high adsorption efficiency, and good reusability for the removal of LEV from aqueous media. The study also discusses the potential of such MIPs as sustainable and efficient tools for addressing antibiotic contamination in water.

## Materials and methods

### Materials

Levofloxacin (LEV) and Gemifloxacin (GEM) were received from Wimits Pharmaceuticals Pvt. Ltd., Lahore. Methacrylic acid (MAA) and ethylene glycol dimethacylate (EGDMA) were purchased from Shanghai Macklin Biochemical Co., Ltd, China. Solvents including methyl alcohol, dimethyl sulfoxide (DMSO) and acetonitrile (ACN), chloroform and acetone were purchased from sigma Aldrich Chemicals, USA. All chemicals and solvents were of analytical grade. Distilled water was used for preparation of solutions.

### Equipment

The qualitative and quantitative measurements of solutions of drugs at various stages of the research work were performed on UV-Vis spectrophotometer (Model Perkin Elmer LAMBDA 25). Fourier Transform Infrared Spectroscopy (FTIR) (Thermo Scientific Nicolet Is10 Infrared spectra) was employed to characterize the molecular imprinted polymers [25] before and after removal of LEV. The surface morphology and elemental composition were investigated on a scanning electron microscope (SEM) (JEOL JSM 6930 LA model) coupled with an energy-dispersive X-ray analyser (EDX). The thermal properties of LEV-MIPs were studied employing a TGA Instrument (Universal Analyser 2000 with Universal V4.7 A software).

### Synthesis of molecularly imprinted polymers

#### Synthesis of LEV-MIPs

For the synthesis of LEV-MIP, the co-precipitation polymerisation technique [26] was employed with slight modification [27, 28]. Moreover, a combination of solvents in various ratios was used as presented in Table 1. A combination of solvents during the development of molecularly imprinted polymers (MIPs) is a well-established approach to enhance the morphology, porosity, and binding efficiency of MIPs [29–31]. Usually, single solvents offer static polarity and intermolecular forces, which may not be suitable to optimise the template–monomer interactions. However, a combination of solvents tends to tune the polarity of the medium and hence establish optimised and stable interactions between the template and functional monomer during the pre-polymerisation stage [32, 33]. A mixture of solvents was taken in a conical flask (250 ml) as presented in Table 1. To this flask, Levofloxacin 0.1mmol (0.036 g) was added as a template. The contents of the flask were solicited for complete dissolution of Levofloxacin in the porogenic solvents (Sonicator: Branson 2510), followed by the addition of 0.678 ml of MAA (methacrylic acid), which plays the role of a functional monomer. Further, the conical flask was gently shaken to make a homogenised solution of Levofloxacin and MAA. After that, 2.97 ml of ethylene glycol dimethacrylate (EGDMA) as cross linker (binds the functional monomer onto the surface of the template) was added to the solution. Finally, an AIBN initiator (0.030 g) was added to the conical flask. Meanwhile, nitrogen gas was purged into the mixture for at least 20 min to remove all the oxygen molecules present in the conical flask. Afterwards, the conical flask was sealed with the aluminium foil tightly to minimise the chances of the addition of steam into the flask during heating on a water bath at 40 °C for the first 5 h and at 60 °C for another 5 h. In this way, jelly-like molecularly imprinted polymers were obtained. LEV-MIPs were filtered and washed with a mixture of methanol and acetic acid. Similarly, the NIPs were thoroughly washed with a mixture of methanol and acetic acid to remove any leftover ingredients. After washing the polymer, allow it to dry in the oven for 48 h. For the synthesis of LEV1-MIP, LEV2-MIP, LEV3-MIP, LEV4-MIP, LEV5-MIP, LEV6-MIP, LEV7-MIP, LEV8-MIP and LEV9-MIP, the complete molar ratio of solvents, template, functional monomer and cross-linking-monomer has been presented in Table 1.

**Table 1:** Composition of imprinted (LEV-MIPs) and Non-Imprinted polymers (NIPs)

MIP	Levofloxacin (mmol/L)	Functional Monomer (MAA)	Cross linker (EGDMA)	Initiator (AIBN)	Porogenic Solvent
LEV1-MIP	0.1mmol (0.036 g)	1 mmol (0.678 ml)	16 mmol 2.97 ml	0.1 mmol 0.03 g	Ethanol: ACN 30 ml:30 ml
LEV2-MIP		2 mmol (1.35 ml)			
LEV3-MIP		3 mmol (1.7 ml)			
NIP1	00	3 mmol (1.7 ml)			
LEV4-MIP	0.1mmol (0.036 g)	1 mmol (0.678 ml)			Ethanol: DMSO 40 ml:20 ml
LEV5-MIP		2 mmol (1.35 ml)			
LEV6-MIP		3 mmol (1.7 ml)			
NIP2	00	3 mmol (1.7 ml)			
LEV7-MIP	0.1mmol (0.036 g)	1 mmol (0.678 ml)			Ethanol: CCl_4_ 40 ml:20 ml
LEV8-MIP		2 mmol (1.35 ml)			
LEV9-MIP		3 mmol (1.7 ml)			
NIP3	00	3 mmol (1.7 ml)			

#### Batch binding assay (rebinding assay)

Nine conical flasks with a capacity of 250 ml each were used. A standard solution of Levofloxacin (20ppm) was added to each flask. Almost 0.5 g of each of polymers labeled as LEV1-MIP, LEV2-MIP, LEV3-MIP, LEV4-MIP, LEV5-MIP, LEV6-MIP, LEV7-MIP, LEV8-MIP and LEV9-MIP was added to each of the above flasks respectively [34]. The above conical flasks were then agitated on an orbital shaker (Vortax Modal-OSM-747) at 150 rpm for six hours continuously. The samples from the above flasks were taken into glass vials after every 30 min time intervals. All the samples were centrifuged for half an hour at 2000 rpm to obtain the MIPs’ free filtrate. The same procedure was repeated for the batch binding of NIPs. The removal accuracy of LEV by the LEV-MIPs and NIPs was recorded by a UV-VIS spectrophotometer at 276 nm ƛ_max_. Extraction efficiency of the samples is represented by Q. The extraction efficiency can be calculated by the following mathematical Eq. (1);

1 $${\text{Extraction efficiency Q }}\left( \% \right)\,=\,{{\text{C}}_{\text{o}}}--{{\text{C}}_{\text{f}}}/{{\text{C}}_{\text{o}}} \times {\text{ 1}}00$$

Where,

C_o_ and C_f_ represent the initial and final concentrations (mg/L) of LEV, respectively.

V is the volume (L) of LEV.

W is defined as the weight (mg) of polymer (MIP or NIP).

### Adsorption parameters

The batch binding efficacy in terms of percentage removal was recorded at adsorption parameters such as contact time, LEV concentration, LEV-MIPs dosage and pH of the solution. The range of all parameters is presented in Table 2. The same protocol was repeated while studying all the parameters used in the rebinding assay.

### Imprinting factor of optimised LEV3-MIP

The imprinting factor (IF) is a significant parameter to ascertain the binding capacity of the MIPs with the template [35]. The magnitude of the IF explains the specific recognition characteristics of a particular MIP and its corresponding NIP to interact with a specific template. The imprinting factor can be computed by following relationship:

2 $$\:\text{I}\text{F}\:\left({\upalpha\:}\right)=\frac{\text{Q}\:\text{M}\text{I}\text{P}\text{s}}{\text{Q}\:\text{N}\text{I}\text{P}}$$

In Eq. (2), Q_MIP_ demonstrates _the_ adsorption capacity of LEV-MIP for LEV, and Q_NIP_ represent _the_ adsorption capacity of NIP for a LEV. Data extracted from batch binding studies were used to calculate the IF of the optimised polymers (LEV3-MIP and LEV6-MIP). Optimised imprinted polymers exhibited an imprinting factor of more than one under given conditions.

## Repeated use of optimised LEV3-MIP and LEV6-MIP

In this study, repeated use of LEV3-MIP and LEV6-MIP after removal of the template (LEV) was computed. Any change in rebinding capacity of LEV-MIPs was noted in ten sequential cycles of LEV adsorption–desorption. These adsorption–desorption experiments were studied under optimised conditions of adsorption parameters.

**Table 2:** Adsorption parameters

Sr. no.	Parameters	Variation in parameter	Constant parameters
1	Initial concentration (ppm)	5,10, 15, 20, 25, 30	Agitation speed 150 rpm, contact time 90 and 60 min respectively, adsorbent dose 0.3 g, pH 7
2	Polymer dosage (g)	0.1, 0.2, 0.3, 0.4, 0.5, 0.6,0.7, 0.8	Agitation speed 150 rpm, contact time 90 and 60 min respectively, pH 7
3	pH	5, 6, 7, 8, 9	Concentration 15 ppm, agitation speed 150 rpm, contact time 90 and 60 min respectively, adsorbent dose 0.3 g

### Selectivity test for optimised LEV-MIPs

The selectivity is usually regarded as the signature property of MIPs. The MIPs are highly selective towards a specific molecule, even in the presence of competitive or interfering molecules in the matrix [36]. The synthesised molecularly imprinted SPR sensors exhibited high sensitivity along with high selectivity for milk samples [37]. MIPs exhibit specific interactions (SI) as well as non-specific interactions (NSI), and hence the selectivity of a MIP can be increased by developing more specific interactions (SI) as compared to non-specific interactions (NSI).

For this purpose, a binary solution of template (LEV) and interferant (GEM) was prepared by mixing 40 ml of each drug having a 10 ppm concentration. Optimised MIPs (LEV3-MIP and LEV6-MIP) were tested against a binary solution for the selectivity test. The distribution ratio (K_D_) between templates (LEV) and LEV-MIPs or NIPs with the solvent was recorded with the help of Eq. (3).

3 $${{\text{K}}_{\text{D}}}={\text{ }}({{\text{C}}_{\text{i}}}--{{\text{C}}_{\text{f}}}){\text{V}}/{{\text{C}}_{\text{f}}}{\text{m}}$$

Where C_i_ and C_f_ stand for original and final concentration (g/ml) of target analyte LEV or interferant GEM in solution, and V presents volume and m represents mass of LEV MIP/NIP used.

The ratio of the K_D_ of Template (LEV) to the K_D_ of interferant (LEV) is known as the selectivity coefficient (K_sel_) given in Eq. (4);

4 $$\:\:{\text{K}}_{\text{s}\text{e}\text{l}}=\frac{\text{K}\text{D}\:\text{T}\text{e}\text{m}\text{p}\text{l}\text{a}\text{t}\text{e}\:\:\:\:}{\text{K}\text{D}\:\text{I}\text{n}\text{t}\text{e}\text{r}\text{f}\text{e}\text{r}\text{e}\text{n}\text{t}\:}$$

Where ‘K_D_ Template’ demonstrates the distribution ratio of LEV-MIP/NIP for analyte LEV and ‘K_D_ Interferent’ shows the distribution ratio of LEV-MIP/NIP for interferent GEM.

The following equation is employed to record the selectivity coefficient (K”)

5 $${\text{K}}\hbox{''}={\text{ }}{{\text{K}}_{{\text{sel}}}}\left( {{\text{LEV}} - {\text{MIP}}} \right)/{\text{ }}{{\text{K}}_{{\text{sel}}}}\left( {{\text{NIP}}} \right)$$

Additionally, the following equation was used to measure the selectivity factor (β) for optimized LEV-MIP:

6 $${\text{b}}\,=\,{{\text{a}}_{{\text{template}}}}/{{\text{a}}_{{\text{interferent}}}}$$

In Eq. (6), α_template_ represents _the_ imprinting factor of template LEV, and α_interferent_ represents the imprinting factor of interferent (GEM).

## Results and discussion

### Synthesis of LEV-MIPs

**Scheme 1:**
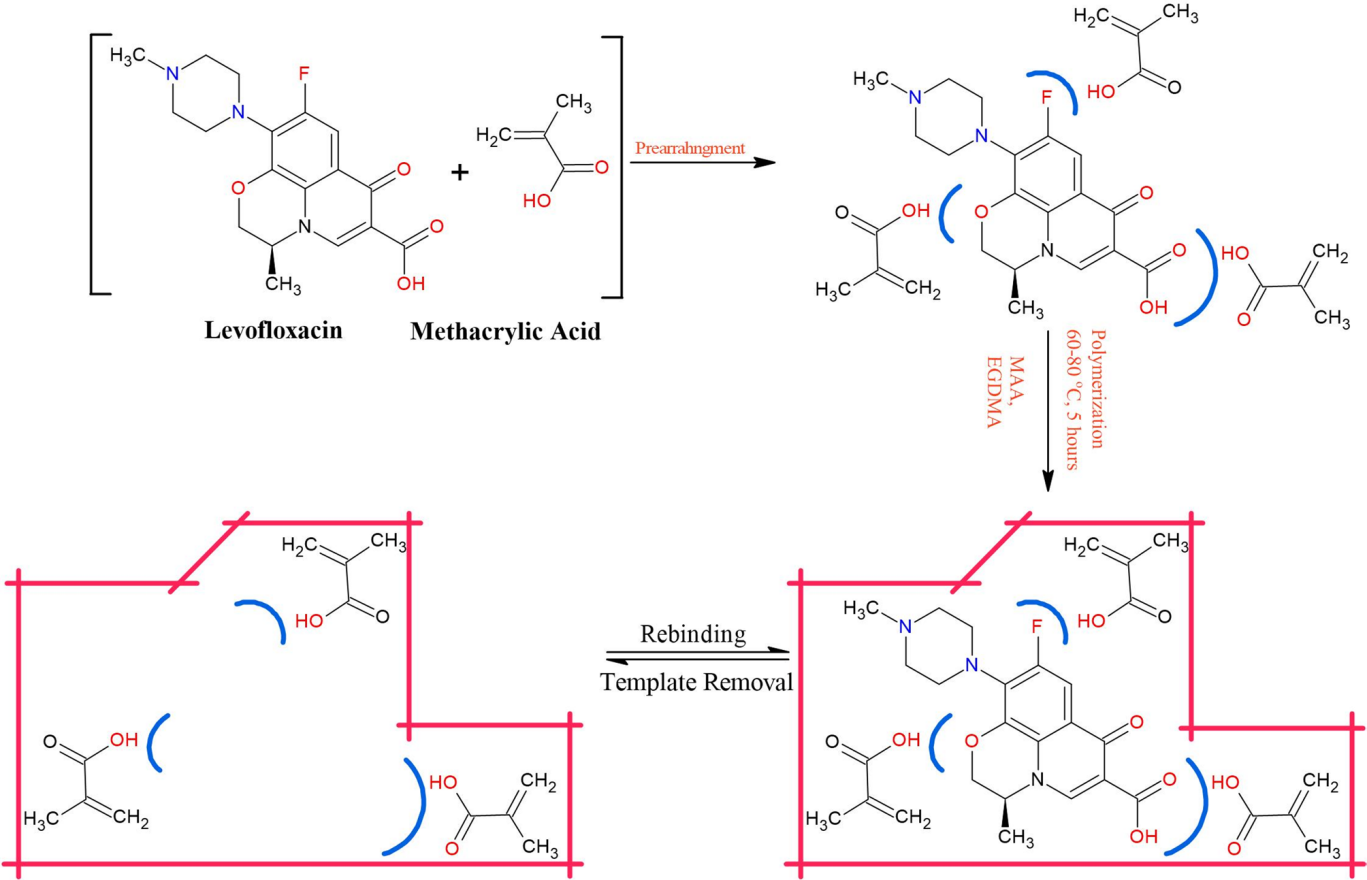
Schematic representation for the synthesis of LEV-MIPs with LEV as a template, MAA as a functional monomer, EGDMA as a cross-linking monomer and AIBN as an initiator

Scheme 1 presents the synthesis of levofloxacin imprinted polymers (LEV-MIPs). Studies illustrated that “chemical imprints” are more effective in the case of average molar masses, low polarity, a small number of ionizable groups and the size of the template [38, 39]. In this study, the LEV bearing average molar mass (361.368 g/mol) consists of carboxylic acid group (–COOH) and piperazine nitrogen as ionizable functional groups. The developed LEV-MIPs have stabilised non-covalent interactions.

During the process of molecular imprinting, weak forces such as electrostatic, hydrophobic and hydrogen bonding were involved between the template-monomer in porogenic solvents before polymerisation. The choice of functional monomer and absolute amount of polymerisation components may lead towards the formation of MIPs offering strong imprinting and extraction abilities. These parameters influence the development and ultimately the efficiency of the MIPs to trap the required template with high selectivity [40]. Studies illustrated that a higher proportion of monomer as compared to template assists the pre-polymerisation of the template-monomer complex. Further, porogenic solvent supports the development of non-covalent interactions between LEV molecules and MAA molecules. It was established that the primary driving force for molecular recognition between MAA and LEV was hydrogen bonding. Carboxylic acid group (–COOH) of MAA developed hydrogen bonding with carboxylic acid, folouro (attached to aromatic ring) and cyclic ether groups of the LEV. Other carbonyl groups and nitrogen atoms of molecules may also assist in interacting with monomer molecules and hence support the fabrication of LEV-MIPs. EGDMA, a cross linker, was used to preserve imprinted cavities of MIP [41] and to maintain the backbone of LEV-MIPs. The prescribed porogenic solvent ratios of ethanol: acetonitrile, ethanol: DMSO and ethanol: CCl_4_ solubilised the constituents and established interactions between LEV and MAA. The used solvent combinations thus control and optimise the distribution of imprinting cavities within the prepared LEV-MIPs [42]. The appropriate combination of porogenic solvents had a prominent impact on the selectivity, surface area and porosity of MIPs [42, 43]. LEV-MIPs and NIPs were obtained in powdered form, having light yellow and white colours, respectively. Afterwards, imprinted polymers were prepared by removal of the LEV (template), which left the impressions related to the size and structure of LEV molecules to actively rebind LEV.

### Fourier transform infrared spectroscopy analysis

**Fig. 1:**
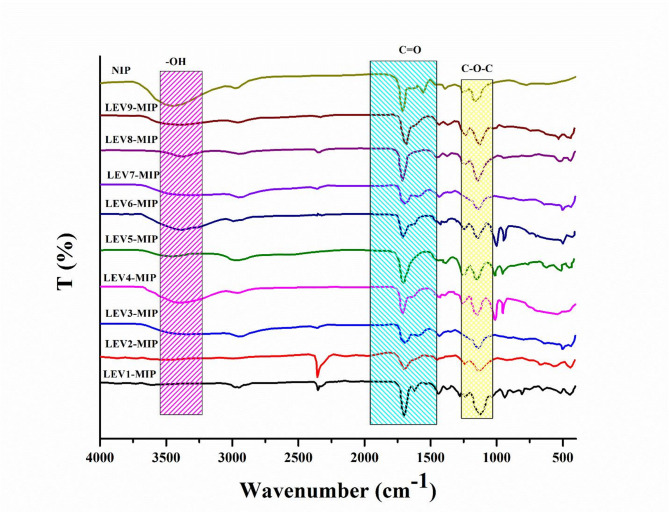
IR Spectra of Unwashed LEV-MIPs1-9 and NIP

**Fig. 2:**
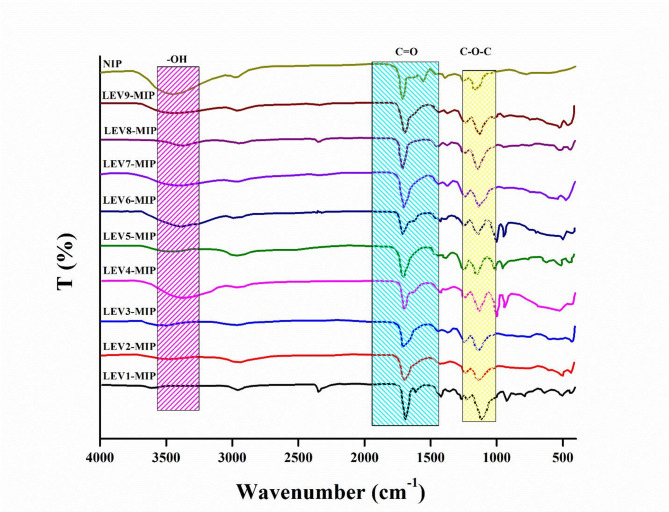
IR Spectra of LEV-MIPs1-9 after washing

FTIR analysis of cross-linked LEV-MIPs was carried out before and after removal of levofloxacin. A comparison of FTIR spectra of a series of LEV-MIPs and NIPs prepared according to the ratios of solvents and functional monomers, as shown in Table 1, has been presented in Figs. 1 and 2.

Spectra of washed and unwashed LEV-MIPs were more or less similar, with only a slight variation in absorption intensity and frequency, highlighting retention of polymeric backbone. All the LEV-MIPs exhibited a broad stretching peak at 3400 cm^− 1^ due to O-H groups present in MAA and LEV molecules. The complementary bending and deformation peaks found at 1388 and 1257 cm^− 1^ endorsed O-H groups. The carbonyl group (C = O) of MIPs and NIP was confirmed by prominent peaks observed at 1725 cm^− 1^. An absorption frequency appeared at 1100, which was attributed to aromatic fluorine, which was prominent in all LEV-MIPs, while this peak did not appear in NIPs, reflecting the absence of LEV in MIPs. The two weak stretching frequencies in the region 2988 and 2951 cm^− 1^ were associated with the symmetric and asymmetric vibrations of the methylene group [36]. The strong peak observed in the region 1638–1640 cm^− 1^ was linked to C = C. The findings of FTIR analysis established the non-covalent interaction of LEV within the MIPs [44, 45].

### Scanning electron microscopy

**Fig. 3:**
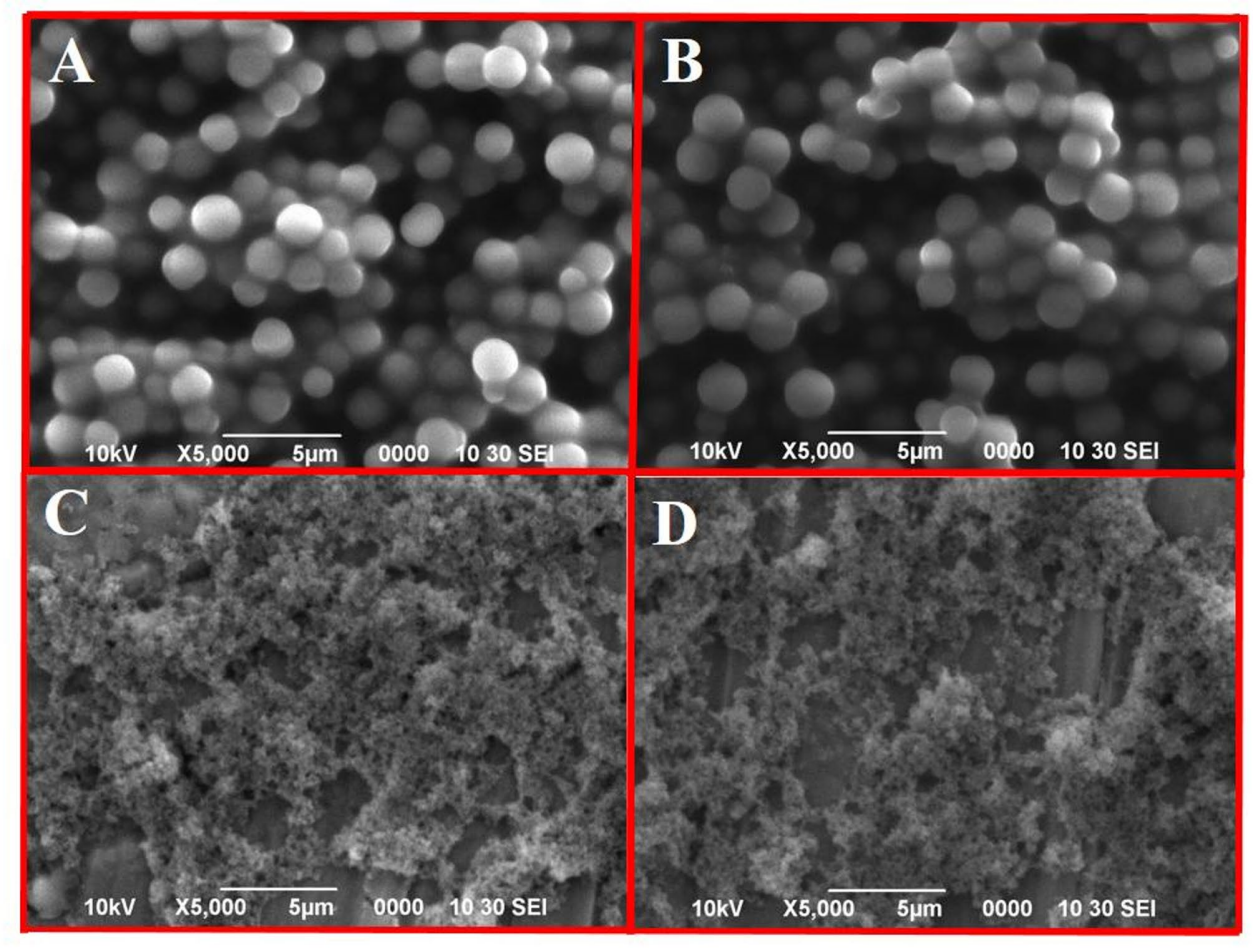
SEM images of LEV3-MIP (A), LEV6-MIP (B), NIP-1 (C) and NIP-2 (D)

The SEM analysis demonstrated that LEV3-MIP and LEV6-MIP exhibited uniform spherical particles (average size 1.5 μm) having smooth surfaces with minimal aggregation, which are indicative of a high degree of cross-linked network formed by ethylene glycol dimethacrylate (EGDMA) as presented in Fig. 3A- B. This kind of morphology favours the consistent diffusion and uniform distribution of the template during the imprinting process. There were no visible imprinting cavities in the SEM images, which does not nullify the molecular recognition potential. In fact, particles having spherical shape and narrow size distribution ensured the efficient removal of template and supports reproducibility and selectivity in exquisite molecular recognition [46]. Both polymers show promise for use in specific adsorption and separation processes involving levofloxacin [47].

### Energy-dispersive X-ray spectroscopy (EDX) analysis

**Fig. 4:**
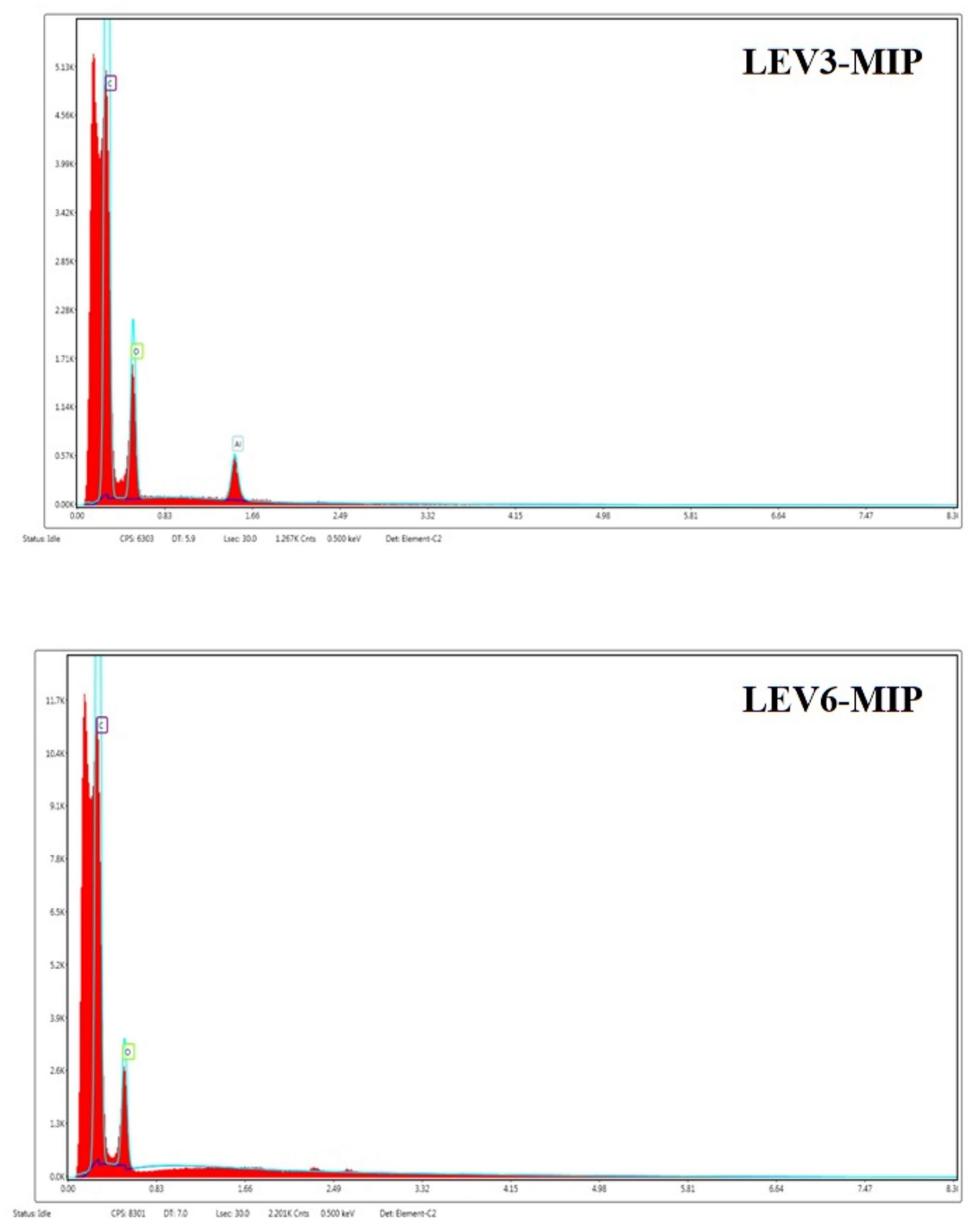
EDX of LEV3-MIP and LEV6-MIP

Elemental composition of imprinted polymers (LEV-MIPs) was analysed by EDX analysis as presented in Fig. 4. The results illustrated the existence of carbon (C), oxygen (O), and nitrogen (N) in both LEV3-MIP and LEV6-MIP, which confirmed the integration of methacrylic acid (monomer) and ethylene glycol dimethacrylate (crosslinker) into the MIP matrix. Higher nitrogen content in imprinted polymers highlighted the entrapment of LEV in the matrix. Both of the MIPs exhibited distinct elemental composition, validating the successful formation of LEV-MIPs. However, LEV6-MIP exhibited a slightly higher proportion as compared to LEV3-MIP, reflecting higher imprinting of levofloxacin and hence better development of recognition sites. Moreover, a well-defined proportion of carbon and oxygen was observed in LEV6-MIP, presenting its structural stability and increased functional monomer interaction.

On the contrary, LEV3-MIP demonstrated a relatively higher oxygen ratio that may be linked to less oxidation or enhanced crosslinking interactions. In addition, traces of fluorine were also observed related to residual levofloxacin residues, which were marginally higher in LEV6-MIP, reinforcing its superior imprinting efficiency.

MIP-LEV6’s elemental composition illustrated that it may present better binding efficiency related to better-organised cavities and optimal imprinting conditions compared to MIP-LEV3.

### Thermogravimetric analysis (TGA)

**Fig. 5:**
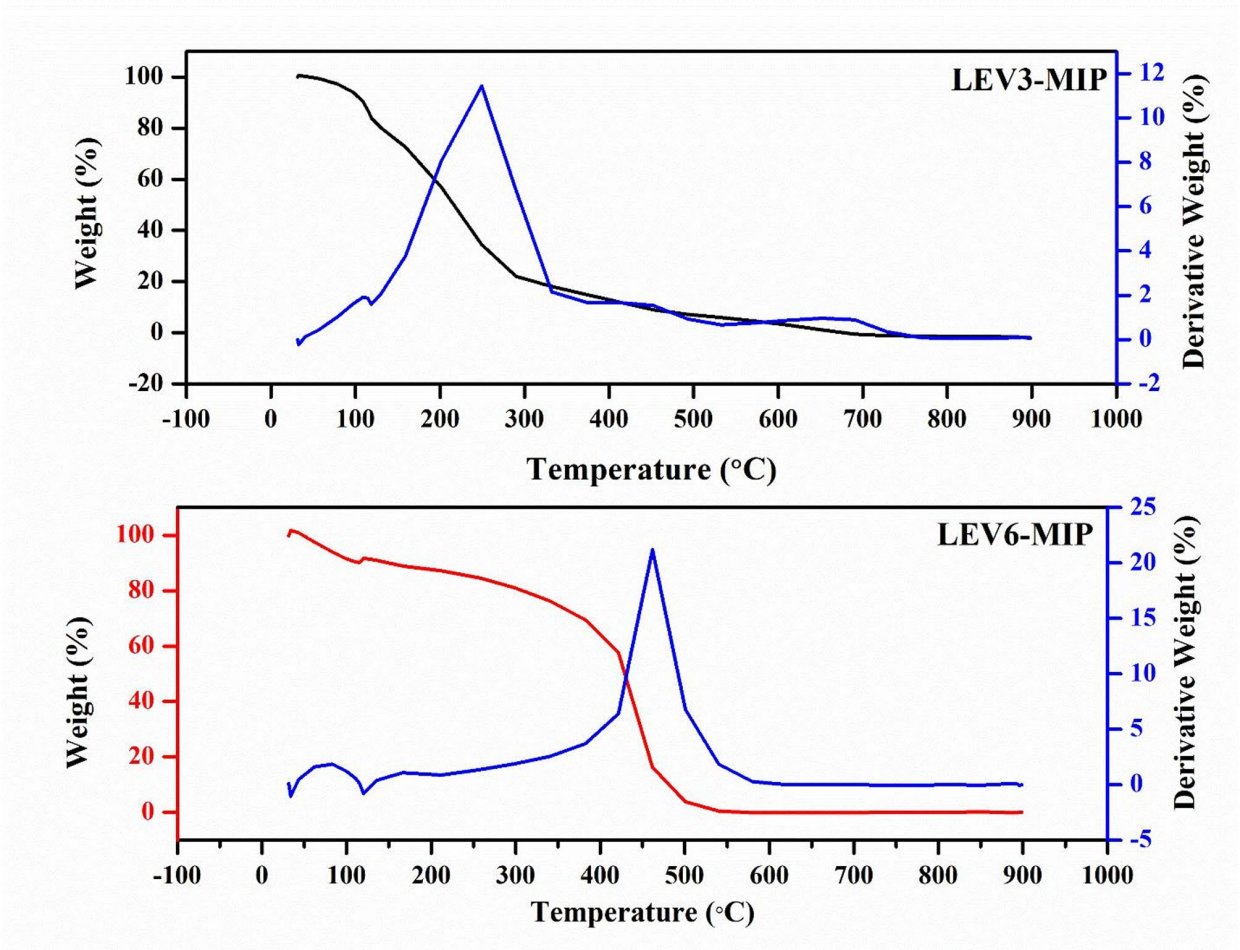
TGA-DTA of LEV3-MIP and LEV6-MIP

TGA was employed to ascertain thermal stability of developed molecularly imprinted polymers (LEV-MIPs) as presented in Fig. 5. Both of the representative LEV3-MIP and LEV6-MIP exhibited initial weight loss observed in the range 50–120 °C, attributed to the evaporation of residual solvents. LEV3-MIP and LEV6-MIP exhibited 15% and 11% weight loss, respectively. Further, major weight loss between 200 and 400 °C occurred due to thermal degradation of the polymer backbone. However, LEV6-MIP had a slightly higher decomposition onset temperature (e.g.,~400 °C) compared to LEV3-MIP (~ 180 °C), indicating better cross-linking density. Moreover, the major degradation peak DTG of LEV6-MIP was sharper and occurred at a higher temperature than LEV3-MIP, suggesting that LEV6-MIP’s network was more robust. LEV6-MIP retained a higher percentage of mass at 500 °C (~ 10%) compared to LEV3-MIP (~ 4%), confirming its enhanced thermal stability. The thermal resistance of LEV6-MIP may be attributed to its optimised cavity structure and efficient interaction between the cross-linking agent (ethylene glycol dimethacrylate) and the functional monomer (methacrylic acid). LEV3-MIP, while slightly less thermally stable, exhibited similar degradation patterns but with slightly lower residual mass, suggesting minor structural weaknesses or less uniform cavity formation. These results validate LEV6-MIP as a superior polymer in terms of thermal resilience, likely enhancing its performance in practical applications, such as drug release or adsorption studies, due to its stable molecular imprinting [24] and robust polymer matrix [48].

### Batch binding assay for LEV-MIPs

**Fig. 6:**
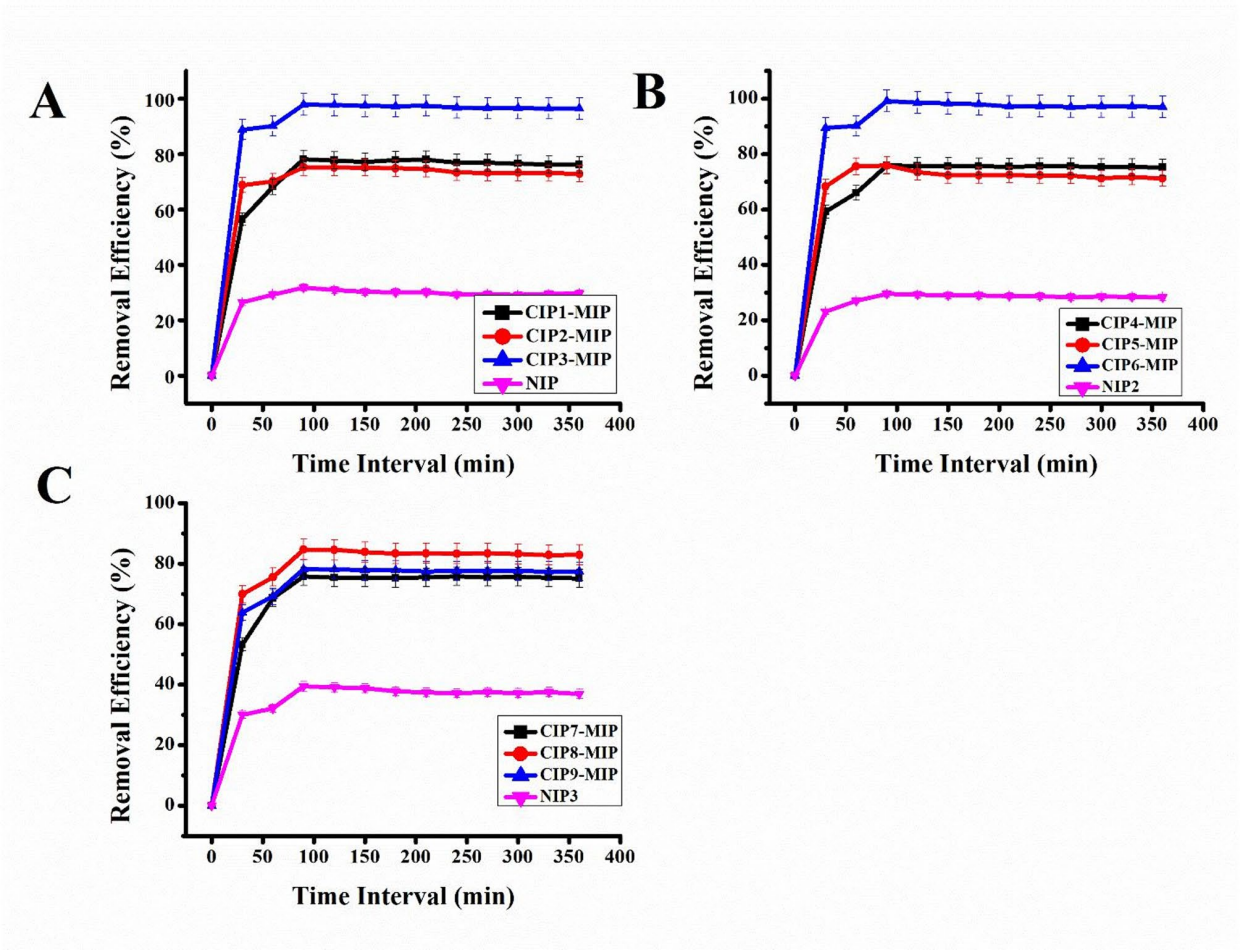
Effect of LEV-MIPs (LEV1-MIP to LEV9-MIP) and NIPs (NIP1 to NIP3) on %age removal of LEV

Batch binding assay was studied using all nine LEV-MIPs (LEV1-MIP, LEV2-MIP, LEV3-MIP, LEV4-MIP, LEV5-MIP, LEV6-MIP, LEV7-MIP, LEV8-MIP and LEV9-MIP) as presented in Fig. 6. The results depicted that the highest percentage removal efficiency was demonstrated by LEV3-MIP and LEV6-MIP. These selected LEV-MIPs were prepared under compatible ratios of LEV (template), functional monomer, and cross-linker, 0.1:3:16 for LEV3-MIP and 0.1:3:16 for LEV6-MIP, respectively. Coincidentally, the ratio of template (LEV), functional monomer, and cross-linker was the same for both MIPs. However, the nature and ratio of the solvent combination were different for each LEV-MIP. This ratio established optimised interactions between levofloxacin and methacrylic acid (MAA), enabling the formation of high-affinity binding sites stabilised by ethylene glycol dimethacrylate (EGDMA) as a cross-linker. The selected combination of porogenic solvents also performed a vital role by improving the solubility and dispersibility while preserving the integrity of the non-covalent interactions during polymerisation. The compatible ratio exhibited more specific recognition sites for the LEV as compared to the other seven LEV-MIP ratios. It was investigated that when the ratio of functional monomer was increased as compared to the template, the non-specific affinity of the imprinted polymer was also increased. NIP-1, NIP-2 and NIP-3 polymers showed minimal removal efficacy due to the absence of recognition sites. For further studies, LEV3-MIP and LEV6-MIP were used due to the highest performance.

### Effect of the contact time

**Fig. 7:**
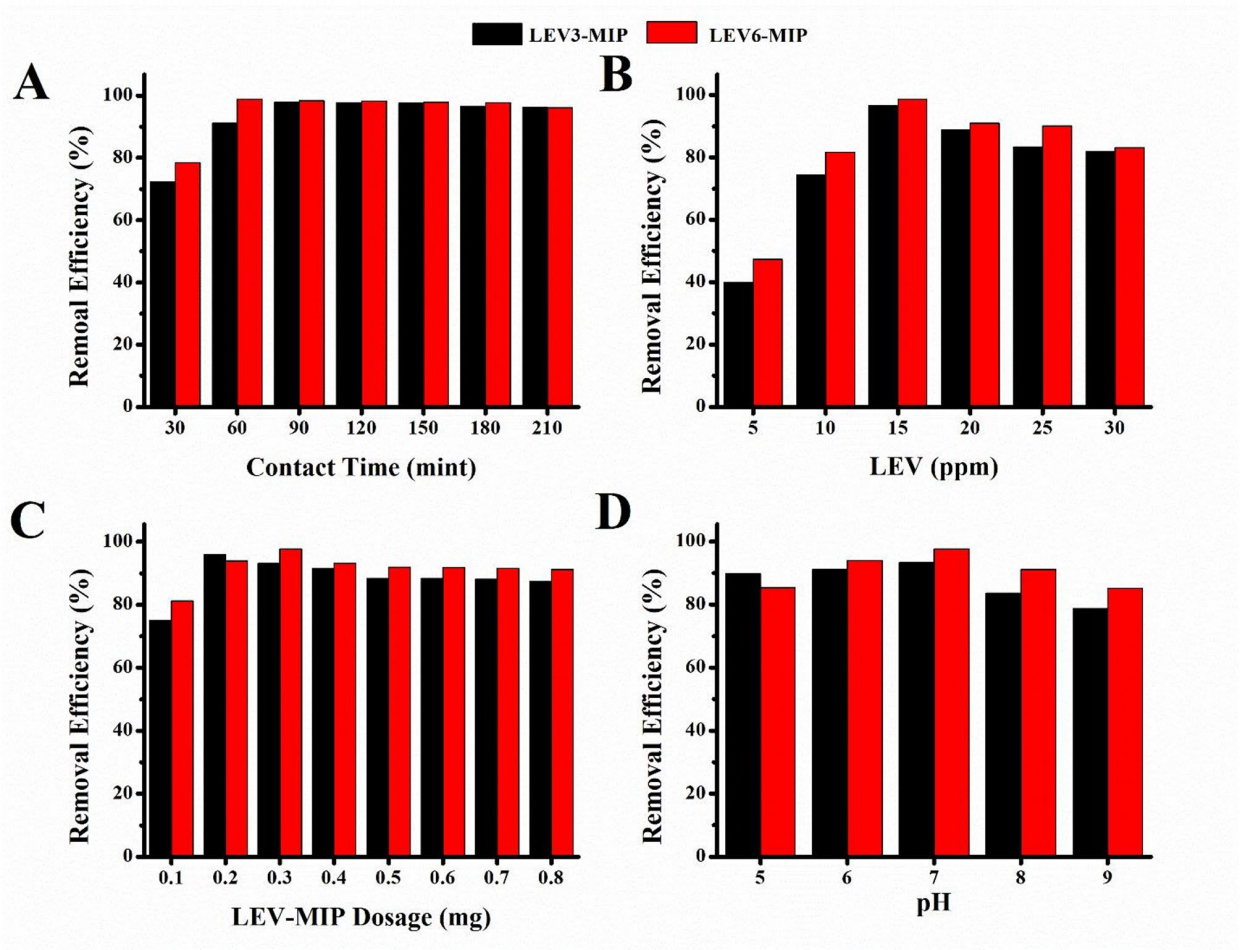
Effect of time (A), Levofloxacin conc. (B), MIP Dosage (C), and pH on % age removal of LEV

It was observed that the removal efficiency of LEV-MIPs increased with the increase in contact time. For this trial, the concentration of the LEV solution was 15 ppm and the MIP dosage was 0.3 g. LEV3-MIP exhibited the highest removal efficiency (97.83%) after 90 min, and LEV6-MIP exhibited the highest efficiency (98.89%) after 60 min, as shown in Fig. 7A. The removal efficiency (% R) of LEV-MIPs was initially increased with the increase in contact time, but further increase in time had no noticeable impact. This is because initially the LEV molecules were attached quickly to MIPs, as the active sites on MIPs were hungry for LEV molecules, and hence maximum removal efficiency was observed. Afterwards, the %age removal of LEV-MIPs reached a dynamic equilibrium. As a matter of fact, the binding cavities of the MIPs were almost saturated with the antibiotic molecules and hence do not permit further adsorption to take place [49, 50].

### Effect of LEV concentration on its uptake behaviour by LEV3-MIP and LEV6-MIP

The effect of LEV concentration on the removal efficiency (% R) of selected MIPs (LEV3-MIP, LEV6-MIP) was studied. As shown in Fig. 7B, the removal efficiency was initially increased with the increasing concentration of LEV solution up to a certain limit. Separate solutions of 5–30 ppm range were taken at a constant contact time of 150 rpm and a constant MIP dosage of 0.3gThe solutions with a higher concentration of LEV have a higher number of target molecules, which in turn surround the active binding sites of the polymer effectively, leading towards a better and efficient adsorption. This trend may also be attributed to the fact that when the concentration of template molecules is higher, the driving forces for mass transfer will be relatively high. For MIP-LEV3 & MIP-LEV6, the maximum % removal efficiency was observed at 96.72% and 98.68%, respectively, with the LEV solution concentration of 15 ppm. Beyond this level of concentration, the % removal efficiency remained almost constant. It is due to the fact that all the binding sites have already been occupied by the target LEV molecules. So, no space was available for the newly incoming molecules of LEV in the polymer cavities [49, 50].

### Effect of LEV3-MIP and LEV6-MIP dosage on the uptake behaviour of LEV

For this study, the concentration of LEV solution and contact time for agitation were kept constant while taking the amounts of the selected LEV-MIPs as a variable. The polymer dosage effect on the adsorption of LEV by LEV-MIPs was expressed in terms of (% R) percentage removal efficiency. Figure 7C shows that the percentage removal efficiency of LEV-MIPs increased with the increase in polymer dosage for representative LEV3-MIPs and LEV6-MIPs up to a certain dosage level, followed by a drop after that.

This study revealed that an increase in the amount of polymer increased the number of binding sites where the target molecules could adsorb. Consequently, the adsorption and adsorption efficiency of the LEV-MIPs increased. It was observed that LEV3-MIP and LEV6-MIP exhibited maximum removal efficiencies of 93.15% and 97.73% respectively, with the polymer dosage of 0.3 g. It was concluded that adsorbent dosages of 0.3 g are the optimum dosages of the selected LEV-MIPs at the optimum time of agitation and at their optimum solution concentrations [50, 51].

Nevertheless, it was observed that when the polymer dosage was increased up to a certain level, its % removal efficiency started to decrease. It was because the increased polymer dosage beyond a certain limit intensified the MIP’s particles. So, MIP particles accumulated into a form of heap, which decreased the number of binding sites available for the LEV molecules. Consequently, the removal efficiency of LEV-MIPs decreased [49, 50].

### Effect of pH on %age removal of Levofloxacin by LEV3-MIP and LEV6-MIP

pH of the solution is a master parameter that greatly affects the batch binning assay. Optimisation of pH is crucial, and its effect on %age removal of levofloxacin by LEV3 MIP and LEV6-MIP was studied from pH 4–9 as presented in Fig. 7D. The study illustrated that developed MIPs exhibited the highest uptake of the LEV (target analyte) at neutral pH conditions. The maximum uptake (93.32%, 97.67%) of levofloxacin was observed at pH 7 by LEV3-MIP and LEV6-MIP, respectively. The batch binding of the template into active sites of MIPs absolutely depends on the nature of functional groups present in template molecules. Moreover, MIPs tend to adopt different conformations at different pH levels, which cause expansion or shrinkage of molecularly imprinted polymers (MIPs). These two factors impact the binding of the template under investigation [52, 53].

In the pH range of 4 to pH 9, molecules of LEV got ionised and were present in the form of a zwitterion. This is due to the ionisation of carboxylic acid and amino groups. At pH 7, both of the functional groups of LEV molecules were active and ready to bind at active sites in the LEV-MIPs cavities. However, in acidic and basic pH, conformational changes in the cavities and ionisation of LEV molecules led to improper “fit into,” which affected the batch binding assay.

### Imprinting factor of optimised LEV3-MIPs and LEV6-MIPs

Imprinting factors (IF, α) are a significant indicator towards definite recognition characteristics of LEV-MIPs for the LEV template with respect to its NIP. IF is related to the ratio of the template (LEV) molecules entrapped in LEV-MIPs cavities versus the template (LEV) attached to the NIP. Higher values of IF stand for stronger interactions of template molecules with MIPs, leading towards higher adsorption capacities. Table 3 shows the IF (α) value for the LEV3-MIP and LEV6-MIP. LEV3-MIP and LEV6-MIPs exhibited a higher IF value (α) of 3.081 and 3.359, respectively, for presenting enhanced degrees of imprinting in them. These noteworthy IF values for LEV-3-MIP and LEV6-MIP are also related to the application of an appropriate polar solvent system; hence, polar solvents are good porogenic solvent compositions for the synthesis of LEV-MIPs [53, 39].

**Table 3:** Study of process parameters on the adsorption/sorption by selected LEV-MIPs

Molecularly imprinted polymer	Process parameters				
	Agitation/ contact time	Concentration	Dosage	pH	IF (α)
LEV3-MIP	90 min	15ppm	0.3 g	7	
	Q = 97.83	Q = 97.83	Q = 93.15	Q = 93.32	3.081
LEV6-MIP	60 min	15ppm	0.3 g	7	
	Q = 98.89	Q = 98.89	Q = 97.75	Q = 97.67	3.359

### Removal of LEV from water samples

The water samples used in the application process were retrieved from, UNIMAS water tank (tap water) and from Sarawak River, Samarahan, Malaysia. The newly synthesised LEV-MIPs were used to remove LEV from water samples spiked with 25 µg/ml of LEV. LEV3-MIP exhibited 23.37 µg/ml (DW), 22.2 µg/ml (tap water) and 21.86 µg/ml (river water) removal of LEV, which was quite higher than that of its NIP, which was only 12.5 µg/ml (DW), 11.7 µg/ml (tap water) and 10.5 µg/ml (river water), respectively, for the same samples. On the other hand, LEV6-MIP exhibited 23.65 µg/ml (DW), 22.8 µg/ml (tap water) and 22.1 µg/ml (river water) removal of LEV, which was quite higher than that of its NIP, which was only 12.75 µg/ml (DW), 11.2 µg/ml (tap water) and 10.8 µg/ml (river water), respectively, for the same samples. The reason is that during the preparation of MIPs, a template was added, which provided multiple types of interactions to the molecules of functional monomer, resulting in greater specificity and affinity for the target analyte [54].

**Table 4:** Removal of CIP from different water samples by using LEV-MIPs and nips

MIPs						NIPs		
	Samples	Amount of LEV added (µg/ml)	Amount of LEV removed (µg/ml)	Recovery %	RSD %	Amount of LEV removed (µg/ml)	Recovery %	RSD %
LEV3-MIP	DW	25	23.37	93.5	0.01	12.5	50	0.02
	Tap Water	25	22.2	88.8	0.03	11.7	46.8	0.01
	River Water	25	21.86	87.44	0.02	10.5	46	0.03
LEV6-MIP	DW	25	23.65	94.63	0.04	12.75	51	0.03
	Tap Water	25	22.8	91.2	0.02	11.2	44.8	0.04
	River Water	25	22.1	88.4	0.03	10.8	43.2	0.01

### Repeated use of optimised LEV-MIP

Recognition ability is one of the outstanding features of MIPs. This ability enables MIPs to re-adsorb specific molecules again, restore their recognition property and maintain their adsorption capacity with minimal change [55]. In order to validate the ability of recognition, ten cycles of adsorption-desorption phenomenon were repeated under optimised conditions of initial concentration of LEV, dosage amounts of LEV-MIPs, agitation time and pH. Experimental data illustrated that LEV-MIPs maintained enough stability during the adsorption–desorption cycles without a remarkable variation in the removal efficiency. Table 4 demonstrates that approximately a 2.7% difference in LEV3-MIP, whereas only a 2.09% difference in LEV6-MIP rebinding efficiency was observed after ten cycles. This negligible loss in extraction efficiency is remarkable evidence of the MIP’s ability to maintain its recognition ability.

**Table 5:** Effect of reused times on the % R of optimised LEV3-MIP and LEV6-MIP

Adsorption–desorption cycle	% *R* (Q) for LEV	
	LEV3-MIP (%)	LEV6-MIP (%)
Cycle-1	97.85	99.10
Cycle-2	97.52	98.72
Cycle-3	97.41	98.41
Cycle-4	97.36	98.21
Cycle-5	97.11	98.07
Cycle-6	97.03	98.02
Cycle-7	97.17	97.50
Cycle-8	97.01	97.32
Cycle-9	96.06	97.14
Cycle-10	95.15	97.01
Overall loss after 10 cycles	2.7	2.09

### Selectivity test for optimised LEV-MIP

The selectivity test is an indicator of the sensing property of LEV-MIPs. Gemifloxacin (GEM) was regarded as a proper interfering and competitive entity for LEV since these drugs exhibit structural resemblance and hence similar physical and chemical characteristics. Selectivity parameters such as distribution ratios (K_D_), selectivity coefficients (K_sel_), relative selectivity coefficients (k′) and selectivity factors (β) were computed using the absorbance data as presented in Table 6. It was established that taking appropriate quantities of solvents and cross linker during the course of precipitation, the polymerisation reaction developed a large number of high-affinity vacant imprinted cavities/sites [56], which had an effect on distribution ratios on the template in MIPs.

**Table 6:** The distribution ratio, selectivity coefficient, relative selectivity coefficient and selectivity factor for optimised LEV3-MIP, LEV6-MIP and NIP

Imprinted polymer	Target	K_D_ (MIP)	K_D_ (NIP)	K^sel^ (MIP) l	K^sel^ (NIP)	K″	Β
LEV3-MIP	Template (LEV)	7.58	0.0777	3.947	1.26	3.13	2.75
	Interferent (GEM)	1.92	0.0613	–	–	–	–
LEV6-MIP	Template (LEV)	19.44	0.0698	3.991	1.14	3.50	3.02
	Interferent (GEM)	4.87	0.0611	–	–	–	–

The results of the batch binding displayed that the distribution ratios of LEV in LEV3-MIP and LEV6-MIP were obviously higher than the distribution ratios of its competitor (GEM). The extraordinary selectivity coefficient factor illustrated that LEV3-MIP and LEV6-MIP are extremely specific for LEV elimination, and the imprinting approach was working accurately. Levofloxacin and Gemifloxacin exhibit similar core structure comprising a bicyclic fused quinolone ring system, carboxylic acid group at position 3, a fluorine atom at position 6 and a piperazine ring at position 7. However, there are some dissimilarities, like levofloxacin has a piperazinyl group at position 1, while gemifloxacin contains a dimethylamino group at position 1. Moreover, levofloxacin has a methyl group at position 7, and gemifloxacin has a methoxy group at position 8, which is not present in levofloxacin. Further, LEV is a relatively smaller molecule compared to GEM, which makes LEV a fit template. Levofloxacin has a piperazine ring at position 1, which makes it a bit flexible, due to conformational changes during binding.

Moreover, LEV is more hydrophilic than GEM. The structural analysis supported that structural compatibility of LEV might present promising interactions with the active binding sites of LEV-MIPs and paved the way for facile entrapment into the cavities as compared to GEM. The presentation of LEV-MIPs illustrated that they are more selective for LEV (template). The distribution ratios of LEV were also found to be higher because LEV-MIPs may recognise and coordinate the LEV molecules by specific active binding sites that have been preserved as a memory [54]. Template (LEV) molecules may easily interact with the comparable cavities present in LEV-MIPs, while an interferent (GEM) presented poor interactions attributed to nonspecific interactions. Additionally, template (LEV) molecules and interferent (GEM) demonstrated more distributions in the LEV-MIPs as compared to NIP because NIP lacks binding sites.

### Limit of detection (LOD) and limit of quantitation (LOQ)

Solutions of Levofloxacin 0f 0.5ppm-8ppm were prepared. Their absorbance was measured by a double beam UV-Vis spectrophotometer by the help of 1 cm quartz cell. A calibration curve was obtained with *n* = 6 levels (triplicate each) having a slope ‘S’ through linear regression. After ten absorbance blank readings, standard deviation (σ) was used to calculate the detection limits as followed by ICH Q2(R1).

$$\:LOD\hspace{0.17em}=\hspace{0.17em}3.3\:*\:/S$$

$$\:LOQ\hspace{0.17em}=\hspace{0.17em}10\:*\:/S$$

By putting σ = 0.078 AU.

S = 0.12 AU µg⁻¹ mL.

The values of LOD and LOQ are obtained as follows.

LOD = 2.1 µg mL⁻¹.

LOQ = 6.5 µg mL⁻¹.

## Conclusion

Levofloxacin imprinted polymers (LEV-MIPs) were successfully prepared and used for batch binding of levofloxacin. Porogenic solvent combinations of ethanol: acetonitrile, ethanol: dimethyl sulfoxide and ethanol: carbontetrachloride were employed to prepare these novel series of LEV-MIPs using a precipitation polymerisation approach. Non-covalent forces were developed between LEV as the template and MAA as the functional monomer by changing the molar ratios of solvents and functional monomer. The outcomes demonstrated that optimised ratio of solvents and functional monomer (0.1:3:16 for template, and functional monomer and cross-linker respectively) prepared LEV3-MIPs and LEV6-MIPs presented percentage removal of about 97.85% and 99.15% of LEV at optimum conditions (contact time 90 and 60 min, polymer dosage 0.3 g, concentration 15 ppm, and pH 7). The FTIR, EDX, SEM and TGA analyses established the successful preparation of LEV-MIPs. SEM images demonstrated that spherical particles having an average diameter (1.5 μm) were monodispersed. Moreover, LEV-MIPs successfully adsorbed LEV as the target analyte from spiked environmental samples. The study established that MIPs are potential adsorbents for the selective and specific removal of an analyte from aqueous media.

## Data Availability

The datasets used and/or analysed during the current study are available from the corresponding author on reasonable request.
